# Supplementary material for: Programmed Cell Death Modifies Neural Circuits and Tunes Intrinsic Behavior
Source: bioRxiv. 2023 Sep 25:2023.09.11.557249. Originally published 2023 Sep 13. Preprint. [Version 2] doi: 10.1101/2023.09.11.557249 (PMC10515839; doi:10.1101/2023.09.11.557249)
Supplement: Supplement 1 [file media-1.pdf]

Table S1. Fates of terminally differentiated relatives of undead cells

Undead Cell	Differentiated Cell Relation	Sister Cell	Niece 1	Niece 2	Grand-niece 1	Grand-niece 2	Grand-niece 3	Grand-niece 4
**ABalaaaalar**	sister	AINL						
**ABalaaaalpa**	sister	ILshL						
**ABalaaaarla**	sister	RMEL						
**ABalaaaarra**	sister	RMER						
**ABalaaaparl**	sister	ILshDL						
**ABalaaapplr**	sister	ILshDR						
**ABalaapaaal**	sister	AINR						
**ABalaapaapa**	sister	ILshR						
**ABalaapapa**	nieces		CEPsoVR	dead				
**ABalaapappa**	sister	CEPsoVR						
**ABalaappaa**	nieces		AVAR	OLQsoVR				
**ABalaapppap**	sister	RIAR						
**ABalaappppap**	sister	IL1R						
**ABalapaaaap**	sister	AVHL						
**ABalapaapap**	sister	RIAL						
**ABalapaappap**	sister	IL1L						
**ABalapapaa**	niece + grandnieces		URYDL		OLQDL	dead		
**ABalapapapap**	sister	OLQDL						
**ABalapappaap**	sister	IL1DL						
**ABalappaaa**	nieces		RID	dead				
**ABalappaapp**	sister	RID						
**ABalappapap**	sister	AVHR						
**ABalapppapap**	sister	OLQDR						
**ABalappppaap**	sister	IL1DR						
**ABalpaaaapp**	sister	m1VL						
**ABalpaaapap**	sister	m2L						
**ABalpaaappa**	sister	MCL						
**ABalpapaapp**	sister	arc ant V						
**ABalpapapapa**	sister	SMBDL						
**ABalpapappa**	sister	SMBVL						
**ABalpappaaa**	nieces		I2L	dead				
**ABalpappaapp**	sister	I2L						
**ABalpapppap**	sister	I1L						
**ABalppaaaa**	nieces		AVAL	OLQsoVL				
**ABalppaapa**	nieces		CEPsoVL	dead				
**ABalppaappa**	sister	CEPsoVL						
**ABalppapppap**	sister	IL1VL						
**ABalpppaapv**	sister	OLLshL						
**ABalpppappa**	nieces		ASKL	dead				
**ABalpppapppp**	sister	ASKL						
**ABalppppaav**	sister	ADLL						
**ABalppppapp**	nieces		OLLL	dead				
**ABalppppapap**	sister	OLLL						
**ABalpppppap**	sister	ASEL						
**ABaraaaapp**	nieces		e1D	dead				
**ABaraaaapaa**	sister	e1D						
**ABaraapapaad**	sister	NSML						
**ABaraapppaad**	sister	NSMR						
**ABarapaaapp**	sister	m1VR						
**ABarapaapap**	sister	m2R						
**ABarapapaaa**	nieces		I2R	dead				
**ABarapapaapp**	sister	I2R						
**ABarapappap**	sister	I1R						
**ABarappaaap**	sister	RIPR						
**ABarappaapp**	sister	hyp1V						
**ABarappapapa**	sister	SMBDR						
**ABarapppppap**	sister	IL1VR						
**ABarpaaapp**	nieces		OLQshDR	CEPshDR				
**CEMDR**	sister	URADR						
**ABarpapaaapa**	nieces		CEPDR	URXR				
**CEMDL**	sister	URADL						
**ABplaaaaapa**	nieces		CEPDL	URXL				
**ABplaapapppp**	sister	ASIL						
**ABplapaaaaa**	nieces		ADEL	ADAL				
**P1aap**	nieces		AVFL	VB1				
**QLaa**	sister	PQR						

Table S1 continued

Undead Cell	Differentiated Cell Relation	Sister Cell	Niece 1	Niece 2	Grand-niece 1	Grand-niece 2	Grand-niece 3	Grand-niece 4
**QLpp**	nieces		PVM	SDQL				
**V5Lpaapp**	sister	PVDL						
**P9aap**	nieces		VA9	VB10				
**P11aap**	nieces		VA11	dead				
**P11aaap**	sister	VA11						
**ABplapapppa**	niece + grandnieces		dead		PLML	ALNL		
**ABplapappppp**	nieces		PLML	ALNL				
**ABplapppaap**	sister	PVQL						
**ABplapppapa**	nieces		HSNL	PHBL				
**TLpppp**	nieces		PHCL	PLNL				
**ABplpaaappap**	sister	OLQVL						
**CEMVL**	sister	AMsoL						
**ABplpaappppp**	sister	CEPVL						
**ABplpappap**	nieces		RMEV	exc cell				
**ABplppaaap**	niece + grandnieces		SIBDL		RICL	dead		
**ABplppaaaapa**	sister	RICL						
**ABplppaapaa**	sister	RIML						
**ABplpppapp**	nieces		PHshL	hyp8				
tail spike	sister	hyp10						
**ABpraaaaapv**	sister	OLLshR						
**ABpraaaappa**	nieces		ASKR	dead				
**ABpraaaapppp**	sister	ASKR						
**ABpraaapaav**	sister	ADLR						
**ABpraaapapp**	nieces		OLLR	dead				
**ABpraaapapap**	sister	OLLR						
**ABpraaapppap**	sister	ASER						
**ABpraapapppp**	sister	ASIR						
**ABprapaaaaa**	nieces		ADER	ADAR				
**Wap**	nieces		AVFR	VB2				
**P2aap**	nieces		VA2	VB3				
**QRaa**	sister	AQR						
**QRpp**	nieces		AVM	SDQR				
**V5Rpaapp**	sister	PVDR						
P10aap	nieces		VA10	VB11				
**P12aap**	nieces		VA12	dead				
**P12aaap**	sister	VA12						
**P12pp**	sister	hyp12						
**ABprapapppa**	niece + grandnieces		dead		PLMR	ALNR		
**ABprapappppp**	nieces		PLMR	ALNR				
**ABprapppaap**	sister	PVQR						
**ABprapppapa**	nieces		HSNR	PHBR				
**TRpppp**	nieces		PHCR	PLNR				
**ABprpaaappap**	sister	OLQVR						
**CEMVR**	sister	AMsoR						
**ABprpaappppp**	sister	CEPVR						
**ABprppaaap**	niece + grandnieces		SIBDR		RICR	dead		
**ABprppaaaapa**	sister	RICR						
**ABprppaapaa**	sister	RIMR						
**ABprpppapp**	nieces		PHshR	hyp9				
tail spike	sister	hyp10						
**MSaaaappa**	sister	m7D						
**MSaaappa**	sister	vpi3D						
**MSaapaapap**	sister	g1AL						
**MSaapapap**	sister	g2L						
**MSapaapa**	M lineage (muscle)							
**MSappapa**	sister	mu bod						
**MSpaaaaap**	sister	M4						
**MSpaapp**	grandnieces				M1	dead	mc3DR	vpi1
**MSpaapaap**	sister	M1						
**MSpapaapap**	sister	g1AR						
**MSpapapap**	sister	g2R						
**MSppaapa**	sister	mu int R						
**MSpppaaa**	Z1 lineage							
**MSpppapa**	sister	mu bod						
**Caapap**	sister	DVC						

Table S2: ***C. elegans*** **strains used in this study.**

Strain Name	Strain Details	Source	Details
N2	*C. elegans* wild isolate	Caenorhabditis Genetics Center	WT
MT1522	ced-3(n717) IV	Caenorhabditis Genetics Center	cell death mutant
IK718	njIs12[glr-3p::glr-1::GFP+glr-1p::RFP+ges-1p::RFP]	Caenorhabditis Genetics Center	RIA label
XE2016	njIs12[glr-3p::glr-1::GFP + glr-1p::RFP + ges-1p::RFP]; ced-3(n717) IV	This paper	cell death mutant, RIA label
XE2852	njIs12[glr-3p::glr-1::GFP + glr-1p::RFP + ges-1p::RFP]; grp-1(tm1956) III; ced-3(n717) IV	This paper	cell death mutant, RIA label, asymmetric cell division mutant
NY2045	ynIs45[flp-15p::GFP] I; him-5(e1490) V	Caenorhabditis Genetics Center	I2 label
XE2019	ynIs45[flp-15p::GFP]; ced-3(n717)IV	This paper	cell death mutant, I2 label
XE2719	ynIs45[flp-15p::GFP] grp-1(tm1956) III ced-3(n717) IV	This paper	cell death mutant, I2 label, asymmetric cell division mutant
IMN31	grp-1(tm1956) III; glt-3(bz34) IV; nuIs5[glr-1::GFP + glr-1::G(alpha)s(Q227L) V + lin-15(+)]V	Caenorhabditis Genetics Center	asymmetric cell division mutant
XE2761	grp-1(tm1956);wdIs52[F49H12.4p::GFP + unc-119(+)]; wpIs116 [F49H12.4p::NLS::mCherry]	This paper	cell death mutant, PVD w/ NLS label, asymmetric cell division mutant
NC1687	wdIs52[F49H12.4p::GFP + unc-119 (+)] II	Caenorhabditis Genetics Center	PVD label
XE1894	wdIs52[F49H12.4p::GFP + unc-119 (+)] II; ced-3(n717) IV	This paper	cell death mutant, PVD label
XE2212	wdIs52[PVD GFP (F49H12.4p::GFP), unc-119(+)] II wpIs119[F49H12.4p::NLS_mCherry::unc-54UTR]	This paper	cytoplasmic GFP and nuclear mCherry in PVDs
XE2213	wdIs52[PVD GFP (F49H12.4p::GFP), unc-119(+)] II ced-3(n717) IV wpIs119[F49H12.4p::NLS_mCherry::unc-54UTR]	This paper	cell death mutant, cytoplasmic GFP and nuclear mCherry in PVDs
OH3191	otIs3[gcy-7p::GFP + lin-15(+)] V	Caenorhabditis Genetics Center	ASEL label
XE1801	ced-3(n717) IV; otIs3[gcy-7p::GFP + lin-15(+)] V	This paper	cell death mutant, ASEL label
XE2760	grp-1(tm1956) III; ced-3(n717) IV; otIs3[gcy-7p::GFP + lin-15(+)] V	This paper	cell death mutant, ASEL label, asymmetric cell division mutant
OH3192	ntIs1[gcy-5p::GFP + lin-15(+)]V	Caenorhabditis Genetics Center	ASER label
XE1802	ced-3(n717) IV; ntIs1[gcy-5p::GFP + lin-15(+)]V	This paper	cell death mutant, ASER label
XE2471	grp-1(tm1956) III; ced-3(n717) IV; ntIs1[gcy-5p::gfp, lin-15(+)] V	This paper	cell death mutant, ASER label, asymmetric cell division mutant
LX836	lin-15(n765ts)X; vsIs44 [tph-1p::GFP] V	Michael R. Koelle Lab	NSM label
XE2001	ced-3(n717) IV; vsIs44 [tph-1p::GFP] V	This paper	cell death mutant, NSM label
XE2820	grp-1(tm1956); ced-3(n717) IV; vsIs44 [tph-1p::GFP] V	This paper	cell death mutant, NSM label, asymmetric cell division mutant
OH15262	otIs669[NeuroPAL] V	Yemeni et al., 2021	NeuroPAL
XE2770	otIs669[NeuroPAL] V; wpIs132[egl-1p::gfp::h2b::egl-1UTR +odr-1p::rfp] X	This paper	Undead cell label and NeuroPAL, cell death WT
XE2769	ced-3(n717) IV; otIs669[NeuroPAL] V; wpIs132[egl-1p::gfp::h2b::egl-1UTR +Podr-1::rfp] X	This paper	Undead cell label and NeuroPAL, cell death mutant
PHX2910	sbIs2910[gcy13p::GCaMP6s + gcy-13p::tagRFP]	This paper	RIM label
XE2718	ced-3(n717) IV sbIs2910[gcy13p::GCaMP6s + gcy-13p::tagRFP]	This paper	cell death mutant, RIM label
XE2738	ced-3(n717) IV sbIs2910[gcy13p::GCaMP6s + gcy-13p::tagRFP] olaEx5053[inx-19p::ced-3a + cex-1p::bfp + unc-122p::rfp]	This paper	cell death mutant, RIM-specific cell death rescue, RIM label
XE2739	wpEx474[inx-1p::rab-3::gfp+ cex-1p::bfp + gcy-13p::glr-1::tagRFP]	This paper	AIB-RIM synaptic label
XE2851	ced-3(n717); wpIs132[egl-1p::gfp::h2b::egl-1UTR +Podr-1::rfp] X; wpEx491[gcy13p::tagRFP]	This paper	cell death mutant, undead cell label with RIM tagRFP label
XE2740	ced-3(n717) IV wpEx474[inx-1p::rab-3::gfp+ cex-1p::bfp + gcy-13p::glr-1::tagRFP]	This paper	cell death mutant, AIB-RIM synaptic label
XE2843	ced-3(n717); wpIs132[egl-1p::gfp::h2b::egl-1UTR +Podr-1::rfp] X; wpEx488[tph-1p::RFP + unc-122p::RFP]	This paper	cell death mutant, undead cell label, extrachromosomal array label of NSM
XE2844	ynIs45[flp-15p::GFP] I; ced-3(n717); wpEx489[tph-1p::RFP + unc-122p::RFP]	This paper	cell death mutant, I2 label, NSM label
XE2845	ces-2(n732ts); vsIs44[tph-1p::GFP] V	This paper	NSM lineage cell death mutant, NSM label
XE2197	ced-3 (n717); wpIs132[egl-1p::gfp::h2b::egl-1UTR +odr-1p::rfp] X	This paper	cell death mutant, undead cell label
XE2198	wpIs132[egl-1p::gfp::h2b::egl-1UTR +odr-1p::rfp] X	This paper	cell death WT, undead cell label
DCR5516	olaIs67 [inx-1p(1 kb)::EGFP::Rab-3 + inx-1p(451bp)::mCherry + unc-122p::RFP];X	*Sengupta* T. et al. 2021	AIB synapse label
DCR5695	ced-3(ola338); olaIs67 [inx-1p(1 kb)::EGFP::Rab-3 + inx-1p(451bp)::mCherry + unc-122p::RFP];X	This paper	cell death mutant, AIB synapse label
DCR4894	olaex2887 [inx-1p(1 kb)::EGFP::Rab-3 + inx-1p(1 kb)::mCherry::PHD + unc-122p::RFP]	*Sengupta* T. et al. 2021	AIB synapse label

Table S3: DNA plasmids used in this study.

Plasmid name	Promoter	Size (bp)	Oligonucleotide 1 (5 → 3)	Oligonucleotide 2 (3 → 5)	Full Construct	Vector
pAK025	egl-1	2756	gctgaaagagtgttgacagtaacc	gacatctccctactatctttcccctata	pegl-1::GFP:H2B::egl-1 3' UTR	pCFJ150
pAK0031	pF49H12.4	2025	gtaagtgagggcaaagtgtgcatc	tcacaacgcacaggtttcac	pF49H12.4::NLS mCherry::unc-54 3' UTR	pDEST R4-R3
pAK0024	pF49H12.4	2025	gtaagtgagggcaaagtgtgcatc	tcacaacgcacaggtttcac	pF49H12.4::mCherry::unc-54 3' UTR	pDEST R4-R3
pAK0044	gcy-13	2000	aaaaattgctaaaagatttataaatcagcaagtggttt	ttgttatttgaaacttattacaaactttcaataatttttcaggac	pgcy-13::GCamP6s::unc-54 3' UTR	pCFJ150
pAK0045	gcy-13	2000	aaaaattgctaaaagatttataaatcagcaagtggttt	ttgttatttgaaacttattacaaactttcaataatttttcaggac	pgcy-13::tagRFP::unc-54 3' UTR	pCFJ150
pAK0047	gcy-13	2000	aaaaattgctaaaagatttataaatcagcaagtggttt	ttgttatttgaaacttattacaaactttcaataatttttcaggac	pgcy-13::GLR-1::tagRFP::unc-54 3' UTR	pCFJ150
pDACR2245	inx-1(1 kb)	1000	attattttctgtgcttttcacaaatacac	tccggcggacaagaac	inx-1p(1kb)::EGFP::Rab-3::unc-54 3' UTR	pSM
pDACR1412	inx-1(451bp)	451	tatagttcttcatcttcttttttttaatatcctc	tccggcggacaagaac	inx-1p(451bp)::mCherry::unc-54 3' UTR	pDEST[4-3]
pDACR2404	inx-1(1 kb)	1000	attattttctgtgcttttcacaaatacac	tccggcggacaagaac	inx-1p(1 kb)::mCherry::PHD::unc-54 3' UTR	pDEST[4-3]
pDACR3149	cex-1	947	ttggaactttaaacgggtttttaaatg	tctagaaatgaacattccatggg	pcex-1::mtagBFP1::unc-54 3' UTR	pDEST[4-3]
pDACR3315	inx-19	5625	acgtaccgaagagatgtg	CAGCAGTTTCCCTGAATTAAA	inx-19p::ced-3A::unc-54 3' UTR	pDEST[4-3]
pDACR489	tph-1	3124	ggtggtcttcccgcttgcaat	gtttttaggtagcattgctctcttcaatcat	tph-1p::tagRFP::unc-54 3' UTR	pDEST[4-3]

H2B: Histone H2B used for chromosomal targeting

NLS: Nuclear localization signal
PHD: Pleckstrin homology domain used for membrane targeting
