# Supplementary material for: Programmed Cell Death Modifies Neural Circuits and Tunes Intrinsic Behavior
Source: bioRxiv. 2023 Sep 25:2023.09.11.557249. Originally published 2023 Sep 13. Preprint. [Version 2] doi: 10.1101/2023.09.11.557249 (PMC10515839; doi:10.1101/2023.09.11.557249)
Supplement: Supplement 2 [file NIHPP2023.09.11.557249v2-supplement-2.pdf]

bioRxiv preprint doi: https://doi.org/10.1101/2023.09.11.557249; this version posted September 25, 2023. The copyright holder for this preprint (which was not certified by peer review) is the author/funder, who has granted bioRxiv a license to display the preprint in perpetuity. It is made available under aCC-BY 4.0 International license.

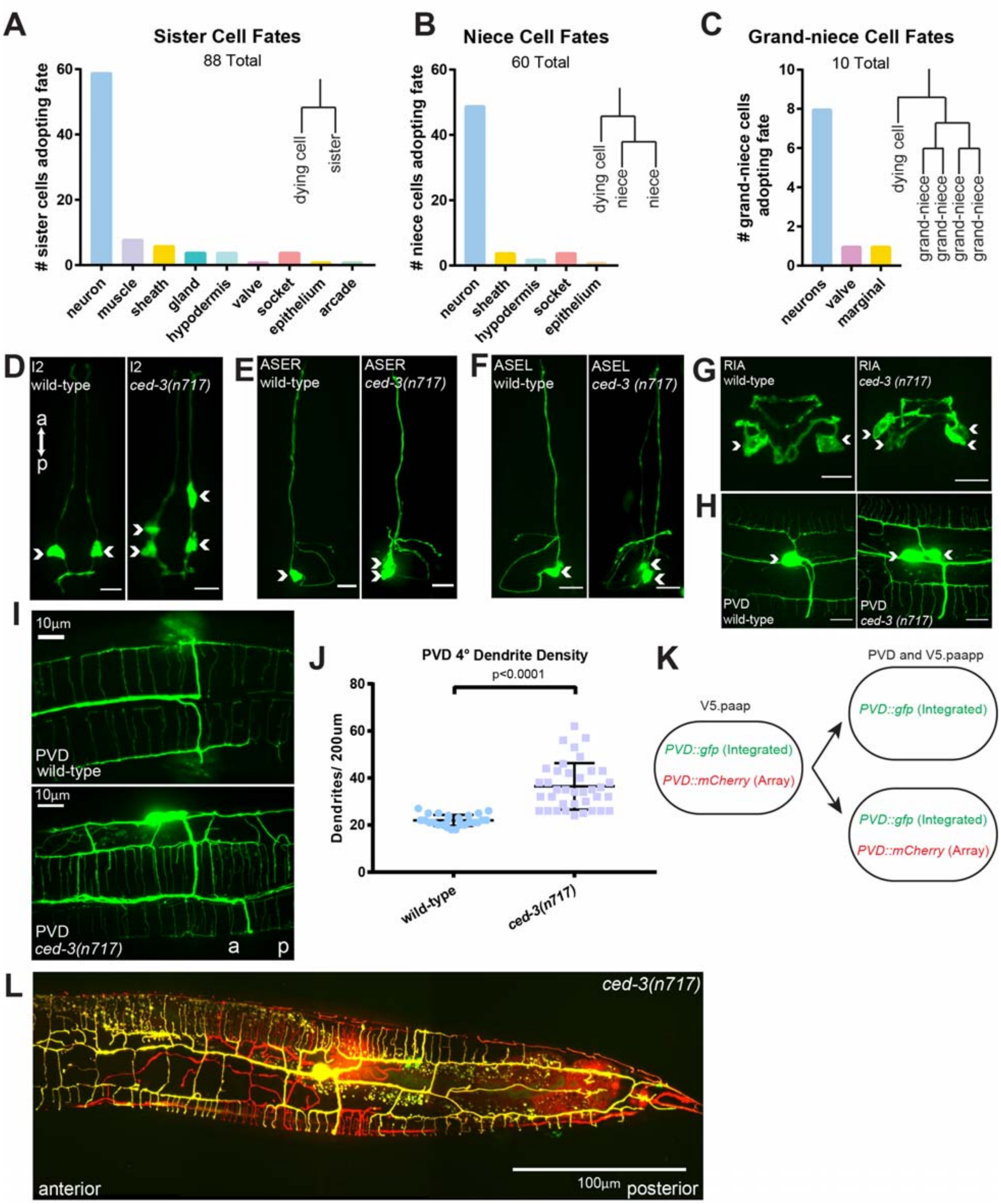

Fig. S1. In *ced-3* mutants, undead cells adopt fates similar to those of their sister cells. (**A-C**) Fates and archetypical lineages of terminally differentiated relatives for all 131 cells removed by PCD in *C. elegans* hermaphrodites. (**D-H**) I2, ASER, ASEL, RIA, and PVD neurons each have sister cells that die in wild-type worms. In *ced-3(n717)* worms, additional cells express GFP markers for each of these neuronal fates (white arrows). 10 µm scale bars. (**I**)

bioRxiv preprint doi: https://doi.org/10.1101/2023.09.11.557249; this version posted September 25, 2023. The copyright holder for this preprint (which was not certified by peer review) is the author/funder, who has granted bioRxiv a license to display the preprint in perpetuity. It is made available under aCC-BY 4.0 International license.

PVD quaternary dendrites viewed on the ventral side of the worm. (**J**) Quantification of quaternary dendrite density in wild-type and *ced-3(n717)* mutants. Data are mean ± S.D. with individual data points shown (wild type, n=26; *ced-3(n717),* n=37). (**K**) Schematic of mosaic PVD labeling. A GFP label is integrated into the genome and inherited by both wild-type and undead PVD cells. An mCherry PVD label is expressed as an extrachromosomal array, which in some animals is inherited by only one of the two PVDs on one side (either the wild-type or the undead cell). This allows visualization of the dendrites from each cell. (**L**) Mosaic PVD labeling demonstrates that both wild-type and undead PVDs produce dendrites.

bioRxiv preprint doi: https://doi.org/10.1101/2023.09.11.557249; this version posted September 25, 2023. The copyright holder for this preprint (which was not certified by peer review) is the author/funder, who has granted bioRxiv a license to display the preprint in perpetuity. It is made available under aCC-BY 4.0 International license.

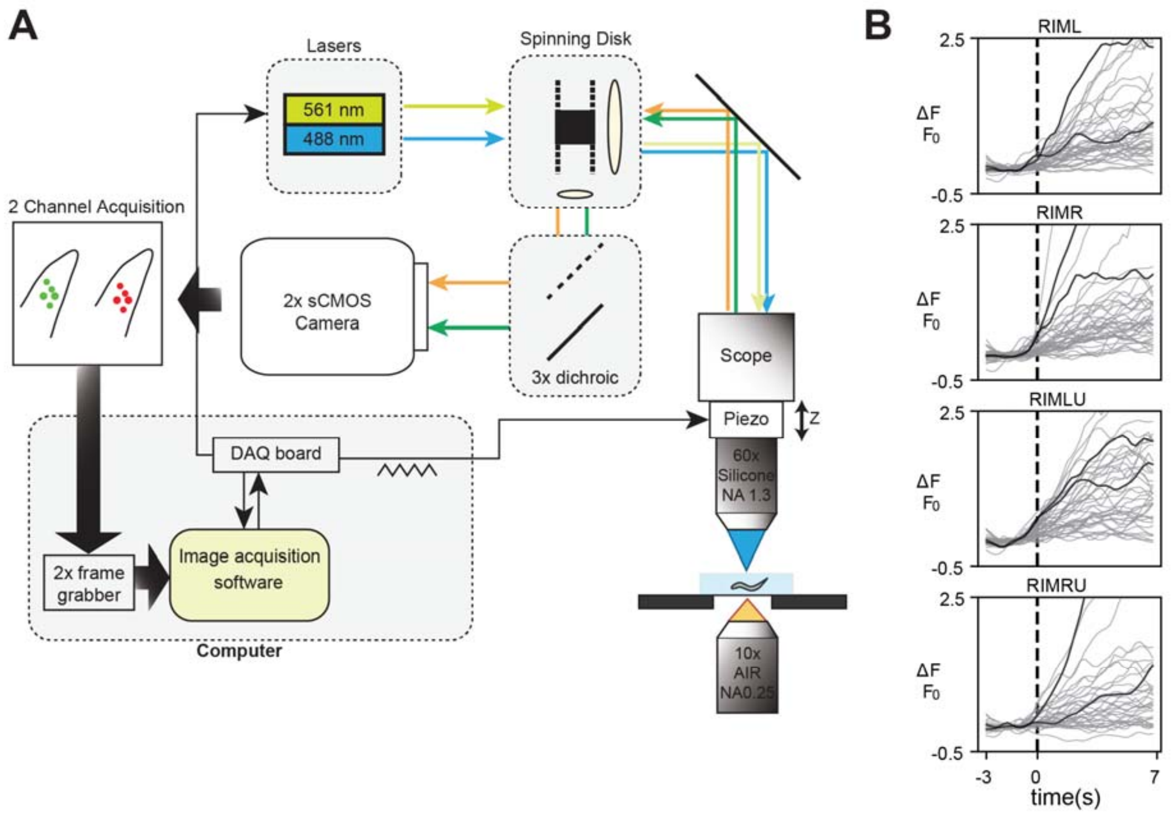

Fig. S2. Function of undead RIM neurons. (**A**) Schematic of calcium imaging. (**B**) Spaghetti plot of all individual traces for average data shown in Fig. 2E. Traces in black are two separate reversal events shown in Fig. 2C.

bioRxiv preprint doi: https://doi.org/10.1101/2023.09.11.557249; this version posted September 25, 2023. The copyright holder for this preprint (which was not certified by peer review) is the author/funder, who has granted bioRxiv a license to display the preprint in perpetuity. It is made available under aCC-BY 4.0 International license.

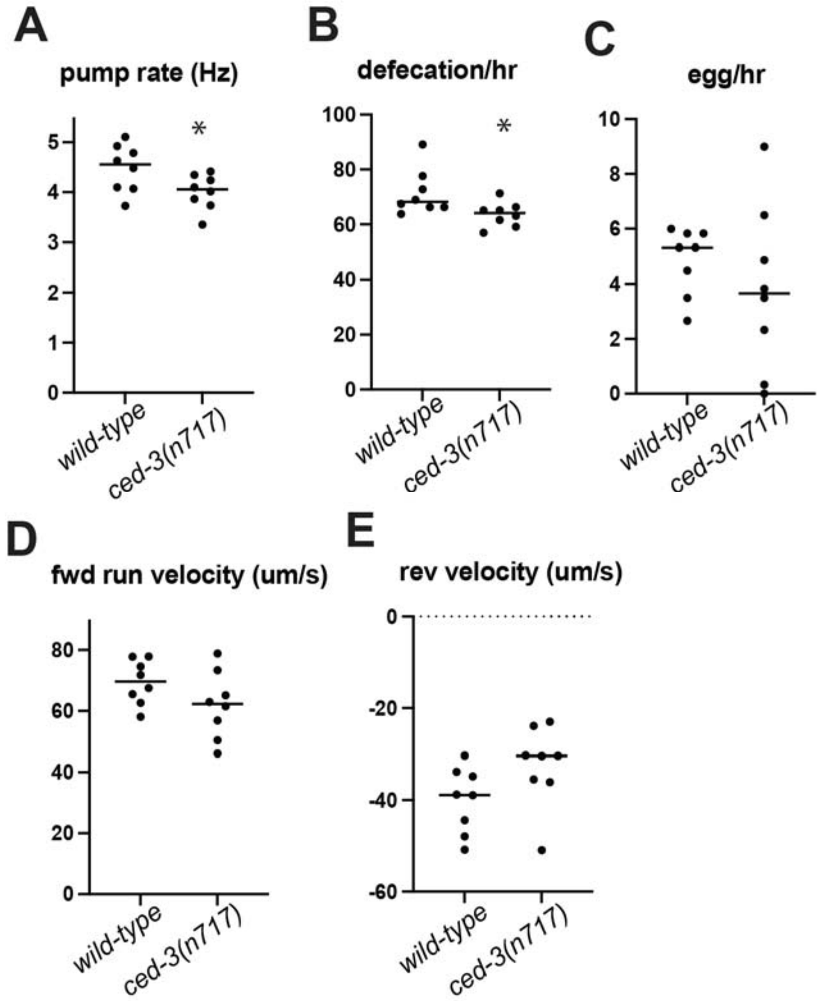

Fig. S3. Additional analysis of *ced-3* behavior. (**A**) *ced-3(n717)* worms have lower pharyngeal pumping rates than wild-type worms. (**B**) Defecation rates are lowered in *ced-3(n717)* animals. (**C**) Egg-laying rate is more variable among individuals for *ced-3* mutant animals. (**D,E**) Forward and reverse run velocity is not significantly different between wild-type and *ced-3(n717)* animals.
